# Rationale for Implementation of Warm Cardiac Surgery in Pediatrics

**DOI:** 10.3389/fped.2016.00043

**Published:** 2016-05-06

**Authors:** Yves Durandy

**Affiliations:** ^1^Perfusion Department, CCML, Le Plessis Robinson, France; ^2^Intensive Care Department, CCML, Le Plessis Robinson, France

**Keywords:** cardiopulmonary bypass, warm perfusion, warm blood cardioplegia, microplegia, pediatric cardiac surgery

## Abstract

Cardiac surgery was developed thanks to the introduction of hypothermia and cardiopulmonary bypass in the early 1950s. The deep hypothermia protective effect has been essential to circulatory arrest complex cases repair. During the early times of open-heart surgery, a major concern was to decrease mortality and to improve short-term outcomes. Both mortality and morbidity dramatically decreased over a few decades. As a consequence, the drawbacks of deep hypothermia, with or without circulatory arrest, became more and more apparent. The limitation of hypothermia was particularly evident for the brain and regional perfusion was introduced as a response to this problem. Despite a gain in popularity, the results of regional perfusion were not fully convincing. In the 1990s, warm surgery was introduced in adults and proved to be safe and reliable. This option eliminates the deleterious effect of ischemia–reperfusion injuries through a continuous, systemic coronary perfusion with warm oxygenated blood. Intermittent warm blood cardioplegia was introduced later, with impressive results. We were convinced by the easiness, safety, and efficiency of warm surgery and shifted to warm pediatric surgery in a two-step program. This article outlines the limitations of hypothermic protection and the basic reasons that led us to implement pediatric warm surgery. After tens of thousands of cases performed across several centers, this reproducible technique proved a valuable alternative to hypothermic surgery.

## Introduction

Perfusion is a typical case of experience-based medicine, and there is no agreement on even such basic factors as priming volume and composition, temperature on bypass, type of cardioplegia and re-dosing intervals, non-pulsatile or pulsatile perfusion. The dogma on the imperative need for hypothermic protection in pediatric cardiac surgery should be discussed in the light of modern anesthesia cardiac surgery and recent cardiopulmonary bypass components. This article aims to present the limitations of hypothermic perfusion and the reasons that led us to implement warm pediatric cardiac surgery.

## Evolution of the Dogma on Hypothermia Protective Effect during Cardiac Surgery

In the 1950s, two major breakthroughs led to the initiation and development of open-heart surgery: systemic hypothermia and cardiopulmonary bypass. In 1950, Bigelow introduced systemic hypothermia with the goal of lowering oxygen requirements, allowing organs exclusion from the circulation for the period necessary for surgery (1, 2). Systemic hypothermia was the only way to obtain a bloodless operating field, allowing intracardiac repair under direct vision. In 1952, Lewis first applied systemic hypothermia (28°C) and a 5.5 min inflow occlusion to close an atrial septal defect. At the same time, the technology of extracorporeal circulation reached the stage of clinical application, and in 1953, Gibbon used the Gibbon-IBM heart–lung machine to close an atrial septal defect. This was the first open-heart surgery with cardiopulmonary bypass. Initially, the two techniques were used separately. In the 1960s, topical hypothermia around 28–29°C with circulatory arrest was mainly used in Novosibirsk in the treatment of ventricular septal defect, atrioventricular canal, and tetralogy of Fallot (3), while cardiopulmonary bypass with normothermia was mainly used in North America (4, 5).

The two innovations were soon combined, and the alteration in systemic temperature was simplified by the addition of a heat exchanger on the bypass circuit (6). Systemic hypothermia was enhanced by topical hypothermia for myocardial protection (7), and by cold cardioplegia in the 1970s (8, 9). There was no real consensus on the optimal temperature needed for the treatment of simple and complex cardiopathies. Moderate hypothermia (30°C) was used very successfully in 337 patients with tetralogy of Fallot (10), but more severe hypothermia was regarded as superior for organs protection allowing an optimal safety margin. Deep hypothermia with circulatory arrest was introduced in the 1960s and later popularized in complex pediatric cases (11–14).

It was commonly admitted that the benefits of hypothermia outweighed its drawbacks, and brain protection during circulatory arrest was indeed a major concern.

The physiology of brain is unique: while this organ accounts for about 2% of the body weight, its blood flow is 13% of the cardiac output, and its oxygen consumption is 20% of the total body oxygen consumption at rest (15). There is no doubt that hypothermia decreases brain oxygen consumption, and it is commonly admitted that this decrease is 6–7% for every degree below 37°C. Consequently, safe periods of circulatory arrest were estimated, but without agreement on the predicted safe duration. From one study to another, it varied from 40 to 60 min for systemic temperature of 20–22°C, to 29 min at 15°C (16–18). This lack of precision was made even worse by the inconsistency of temperatures recorded at various sites. In a study using electroencephalogram assessment of electrocerebral silence as an objective measure of brain function, the authors demonstrated that temperatures from nasopharynx, esophagus, and rectum were inaccurate in predicting the cessation of brain electrical activity. Electrocerebral silence was observed with nasopharyngeal temperatures varying from 10.1 to 24.1°C and rectal temperatures varying from 12.8 to 28.6°C (19). The safe duration of circulatory arrest is not the only uncertainty in the management of deep hypothermia and the choice between pH-stat versus α-stat strategy is an endless debate (20–26). In the past, despite hypothermic brain protection, neurologic complications were frequent. Postpump chorea was strongly associated with deep hypothermia and circulatory arrest and the prognosis was guarded (27, 28). Much more frequent were post circulatory arrest clinical seizures, with an incidence of around 6–10% (29, 30) but three times more when seizures were diagnosed *via* continuous electroencephalographic monitoring (31). There is some evidence that neonatal seizures are a good surrogate marker of long-term neurologic outcome (32). As survival rate improved for congenital heart surgery, long-term neurodevelopment delays were observed in patients treated with deep hypothermia. The dogma of brain hypothermic protection during circulatory arrest became progressively discussed, and antegrade selective cerebral perfusion was introduced to overcome the side effects of circulatory arrest with profound body hypothermia (33–35). The term cerebroplegia was used for cerebral perfusion with 6–10°C blood, while the body was perfused with moderate systemic cooling of around 26°C. Pediatric surgeons accepted to implement this technique for hypoplastic left heart syndrome first stage palliation or aortic arch surgery (36, 37). The benefits were disappointing compared to circulatory arrest, and several works failed to find any positive effect of antegrade cerebral perfusion (38–41). The appropriate hypothermic brain perfusion rate and pressure for neonates remain unknown. Many studies assessed hypothermic perfusion using oxygen delivery or oxygen saturation as a marker of perfusion quality. In fact, high oxygen saturation during hypothermic perfusion may indicate impaired oxygen transfer from blood to tissue rather than normal or over-perfusion (42). Besides, optimizing oxygenation does not mean “the more, the better,” and any excessive blood flow can be detrimental, carrying the risk of cerebral edema and intracranial hemorrhage (43).

The mechanisms of hypothermic brain protection are still to be elucidated and decades after its implementation, the management of patients during hypothermia and rewarming is far from uniform. This is, at least in part, due to the lack of clear evidence about the optimal management.

The physiology of the heart is quite unique too. Being a muscle, its myocardial oxygen consumption is mainly related to its activity. At rest, the coronary blood flow represents 5% of cardiac output, while its oxygen consumption is 11% of the total consumption (15). Hypothermic myocardial protection during cardiac surgery is more questionable, as hypothermia increases oxygen consumption per beat, the decrease in oxygen consumption observed being due to bradycardia. It has been estimated that myocardial oxygen consumption, normalized for heart rate, more than doubles when temperature decreases from 37 to 22°C (44, 45). Furthermore, the decrease of myocardial oxygen consumption of an arrested heart is little influenced by temperature and, in dogs, there is an overlapping between myocardial oxygen consumption of a normothermic contracting empty heart and a heart during hypothermic (11°C) arrest (46).

Hypothermic protection of other organs including kidney, liver, gut, lungs, muscle, skin, and endocrine glands is even more questionable. Hypothermia depresses renal blood flow, glomerular filtration, osmolar clearance, and maximum tubular excretory capacity (47, 48). Glomerular filtration rate and renal plasma flow decrease by 40% at 32°C and 70% at 27°C (49). The blood flow of liver, gut, and pancreas is reduced during hypothermia, with a decrease in liver metabolic and excretory functions. There is some concern about the role of hypothermia in the increase in gut permeability and loss of mucosal integrity observed following cardiopulmonary bypass. These gastrointestinal changes underline the risk of endotoxin or bacteria translocation from the gut to the blood (50, 51). The mechanical effects of hypothermia on lungs have been studied in sheep. A significant decrease in compliance was observed in animals subjected to hypothermia, while compliance was stable in animals subjected to anesthesia alone or normothermic bypass (52). The effects on soft tissue were rapidly established, as the first case of lethal subcutaneous fat necrosis was published in 1953 in a four-and-a-half-month-old boy operated on for a tetralogy of Fallot (53). A few cases of this rare complication have been published with a more favorable evolution (54). Endocrine function is altered during hypothermia, the most frequent manifestation being a decrease in insulin production with hyperglycemia (55).

Apart from organ dysfunction, reversible platelet dysfunction (56), depressed immune system (57), and pH and PCO_2_ modifications (58) have been related to hypothermia. There is also reasonable suspicion that increased endothelial permeability and edema may contribute to organ malperfusion during cooling and rewarming (59, 60).

The side effects of hypothermia are all reversible, and there is no doubt that hypothermic perfusion has been essential to the development and success of cardiac surgery. However, following hypothermia, the delay between normalization of temperature and normalization of organ function and metabolic drawbacks is likely to vary from one case to the other. This is particularly true for neurologic impairment, which may even persist in grown-up children (61), but possibly also for immune function, contributing to a higher risk of postoperative infection (62), and coagulation impairment, with its consequences (bleeding and transfusion) carrying their own risk (63, 64).

## Brain Warm Ischemia: A Misunderstood Risk

A common opinion is that during normothermic, 5- to 10-min circulatory arrest, brain changes and neuronal death are irreversible. This is not totally in agreement with the observations in dogs, which can stand up to 30 min of circulatory arrest without permanent brain damage, provided blood is removed from the brain before the arrest (65), nor with observations made on humans following prolonged circulatory arrest. In such cases, hypoxia should affect all the brain with symmetrical destruction while, in fact, the destruction of large areas on one side co-occurs with unaffected corresponding areas on the opposite side (65). This is not in agreement with a recent experimental work on pigs, demonstrating that brain recovery following 30-min warm ischemia depends on the quality of reperfusion. The poorest outcomes, including brain edema and extensive cerebral infarct, were observed with “uncontrolled reperfusion,” while pulsatile “controlled reperfusion” resulted in minimal brain edema and no brain infarction. Uncontrolled reperfusion was defined as normal blood reperfusion by the pig heart, and controlled perfusion was performed *via* a mechanical pump with a high flow (66). This experimental work could contribute to explain the superior results observed with extracorporeal membrane oxygenation rescue versus conventional cardiopulmonary resuscitation. In one work on in-hospital cardiac arrest in pediatric patients, 11/27 patients (41%) were survivors, 10 of them having good neurologic outcomes (67).

There is, of course, no study establishing the safe limit of brain warm ischemia in man, and data have only been gathered from experience. Unexpected recoveries were observed following treatment of massive hemorrhage with deliberate circulatory arrest using induced ventricular fibrillation. Normothermic arrests up to 14 min were observed without sequelae (68). There is some evidence that the time to irreversible brain damage following normothermic brain ischemia is not necessarily as short as commonly admitted. Ischemia–reperfusion effects could be influenced by the quality of reperfusion and anticoagulation avoiding thrombosis in small brain vessels during circulatory arrest.

During full flow warm oxygenated blood perfusion, brain perfusion is controlled by auto-regulation. In case of an incident requiring circulatory arrest longer than the “classical” safe limit, controlled reperfusion should be used to obtain full recovery of the neurologic function. The new challenge is therefore to compare the inherent risks of hypothermic brain perfusion or hypothermic circulatory arrest against the inherent risks of warm perfusion.

## Warm Cardiac Surgery, the Adult Experience

Continuous whole body warm perfusion with oxygenated blood is one way to avoid the deleterious effects related to ischemia–reperfusion. This option, used in the early years of cardiac surgery (4, 5), was then abandoned until 1989, when a 64-year-old woman was operated on for mitral valve surgery with 33°C perfusion and 37°C continuous cardioplegia infusion. Due to cardiac rupture, the cross clamp time was 393 min. This patient was easily weaned off bypass without inotropic support or intra-aortic balloon pump, but died 17 h later from recurrence of arterio-ventricular separation (69). Following this impressive result, a randomized study on 1,732 coronary bypass surgery patients, classified into warm group (*n* = 860) and cold group (*n* = 872), failed to demonstrate any advantage of hypothermic brain protection, and serial creatine kinase MB fraction levels were significantly lower in the warm group (70, 71).

The constraint imposed by continuous cardioplegia infusion, be it antegrade or retrograde, was overcome in 1995. Intermittent antegrade warm blood was compared to cold blood cardioplegia in two groups of coronary artery bypass graft patients. The warm group showed improved outcomes in term of immediate hemodynamics and peak concentration of creatine kinase myocardial-specific isoenzyme. The incidence of myocardial infarction and stroke was lower in the warm group, albeit not reaching significance (72, 73). Warm perfusion and intermittent warm blood cardioplegia was implemented by other groups, confirming the feasibility, safety, and benefits of the technique (74–77). A meta-analysis failed to demonstrate any difference related to perfusion temperature in the incidence of stroke and deterioration of the neuropsychological function following cardiopulmonary bypass (78). The lack of difference between cold and warm perfusion could be due to the fact that the period at higher risk of embolization occurred at the beginning and the end of bypass, when patients of both groups were normothermic. It is noteworthy that warm adult surgery, introduced 20 years ago, is used worldwide and that the risk of bypass circuit mechanical incident-related brain ischemia is more than exceptional. Intermittent warm blood cardioplegia was mainly used in coronary artery bypass graft and re-dosing intervals of 15 min or less were more than sufficient to perform a distal anastomosis. However, there was some concern regarding the maximum safe time to re-dosing. In the literature, the safe time to re-dosing increases progressively with increased experience in warm surgery. It was 5 min in 1992 (79), 10–15 min in 1995 (73–75), 30 min in 2000 (80), or even more. Single shot warm blood cardioplegia was used for aortic clamp time between 29 and 47 min (81).

## Implementation of Warm Pediatric Surgery a Two-Step Shift

We found several aspects attractive in warm perfusion and with recent oxygenators circuits and cannula, in experienced hands, the need for circulatory arrest was no longer essential. In the early 1990s, we thus shifted from cold to warm perfusion. Continuous warm blood cardioplegia being unrealistic in pediatric patients, the first step of our protocol combined warm perfusion with cold blood cardioplegia. The prime was heated to 37°C and the heater–cooler unit was set to 37.5°C during the whole bypass time. Such a protocol was not totally satisfactory, but we learned that warm perfusion was feasible and safe and observed a number of positive outcomes. As expected, time on bypass, a classical risk factor in pediatric cardiac surgery, decreased. The endless debate on the best pH management during hypothermic perfusion was avoided, as well as the risk of hyperthermic brain injury during rewarming (82). Convulsion, a manifestation usually related to deep hypothermia, was no longer observed, preventing long-term neurological impairment (32, 83, 84), and myocardium rewarming between cardioplegia re-dosing was limited by the interposition of a mattress perfused with cold water.

When intermittent warm blood cardioplegia was proved to be reproducible, efficient, and safe in the adult population, we implemented this technique in pediatrics. The second step combining warm perfusion and cardioplegia started in April 2001. Warm blood cardioplegia was in fact microplegia. Warm oxygenated blood was diverted from the oxygenator or from the origin of the arterial line *via* a roller pump. Downstream the roller pump, the arresting agent was added *via* an electrical syringe pump. The ratio of blood to arresting agent was 60:1 and the cardioplegia was sucked back into the circuit. The hydric balance of microplegia was negligible, limited to the few milliliters of arresting agent (85). The arresting agent was similar to St. Thomas I, which is composed of potassium, magnesium, and procaine. The re-dosing interval was 15 min, and we did not observe any hyperkaliemia. Our initial experience with this new protocol was satisfactory. We compared retrospectively the last 950 patients operated on with warm perfusion and cold blood cardioplegia and 1,400 patients operated on with warm perfusion and intermittent warm blood microplegia. Spontaneous resumption of sinus rhythm was 99% in the warm group versus 77% in the cold group, and intensive care length of stay was under 48 h in 86 versus 75%. Four groups of frequent cardiopathies were selected to compare time to extubation and postoperative troponin levels: ventricular septal defect, tetralogy of Fallot, complete atrioventricular septal defect, and arterial switch operation. In each group, the results were significantly enhanced following warm surgery. Among the selected patients, mortality was comparable: 5 out of 364 (1.4%) in the warm group versus 5 out of 255 (1.9%) in the cold group. Following this study, we compared risk stratification in our unit to risk assessment from the Aristotle basic complexity score. Patients under 10 kg were included in the study, 38 had prolonged aortic cross-clamp time defined as a cross clamping time exceeding 90 min (group 1), and 196 had shorter cross clamp time (group 2). In terms of mortality and prolonged hospital length of stay, our results compared favorably to the data from the Society of Thoracic Surgeons and European Association for Cardio-Thoracic Surgery database. Interestingly, blood lactate level, a biomarker of perfusion quality, was at 2.5 mmol during bypass, peaked at 2.6 mmol/L on arrival to ICU in group 1 and reached 1.4 mmol/L 20 h later (86). This level was far lower than those associated with complicated postoperative courses described in the literature (87, 88).

The results obtained with warm surgery in our unit proved reproducible. The benefits of warm perfusion on immediate outcomes were confirmed through low requirement for inotropic and short time to extubation, low lactate production, adequate urine output, minimal drainage from the chest drains, short ICU and hospital stay (89). When compared to hypothermic perfusion, warm perfusion was associated with reduced oxidative stress (90). A study comparing arterial switch operation with either cold or warm surgery confirmed the benefits of warm perfusion on postoperative lactate blood levels, time to extubation and length of stay in ICU (91). Myocardial protection assessed on myocardial biopsies confirmed the superiority of warm blood microplegia, while early and late neurodevelopmental status, following warm perfusion, were equivalent to those observed during mild hypothermia (92). The absence of benefits of low temperature over warm perfusion on brain protection became ever more evident. Of note, several European units switched from cold to warm surgery, and none decided to shift back to hypothermia (89–94).

The growing experience in intermittent warm blood microplegia consolidated a significant body of evidence to support the good tolerance of warm myocardial ischemia. The issue was, as it has been for adults, to determine the maximal safe time to re-dosing. We progressively increased time to re-dosing from 15 to 35–40 min without any drawbacks (95–97). This slow drift was done over 10 years and was due to the frequent “2 min” delay requested by the surgeon. In a study assessing Troponin T following 35–40 min re-dosing intervals, we compared 4 groups of patients: 46 patients had cardiac surgery without aortic cross clamping (group 0), 81 patients had one cardioplegia injection (group 1), 31 patients had two injections (group 2), and 7 had 3 injections (group 3). There was no significant difference in Troponin T levels between group 0 without myocardial ischemia and group 1 with 28.30 ± 8.84 min cross clamp time. In group 2 (65.71 ± 9.70 min cross clamp time), troponin was 2.17 ± 2.29 μg/L and in group 3 (114 ± 13.71 min cross clamp time), the level was 3.79 ± 3.00 μg/L. These blood levels of troponin compare favorably to those from patients receiving cold crystalloid cardioplegia (87, 88, 98). The main limitation of our study is the small number of long-term aortic cross clamp time.

Twenty years from the implementation of warm perfusion, and 15 years after we shifted to warm surgery, thousands of cases have been performed every year, mainly in Europe. The safety, efficiency, and advantages are widely acknowledged by surgeons who adopted this approach. We have been using the technique for all types of cardiopathies, including interruption of the aortic arch without or with transposition of the great arteries or total pulmonary anomalous venous return. During aortic arch repair, the arterial line was divided in two *via* a Y-type connector. The upper part of the body was perfused through the brachiocephalic artery and the lower part through the descending aorta or the femoral artery. Small femoral cannulas were specifically designed for neonates. We did not experience any of the drawbacks of femoral cannulation and immediate postoperative arterial Doppler demonstrated the permeability of the femoral artery. Many surgeons are less enthusiastic and prefer the comfort of circulatory arrest for complex cases. There is no discussion about the fact that cardiac surgery is teamwork and that the quality of surgical cure is of utmost importance. The final decision in the operating room belongs to the surgeon. However, warm surgery is not a “point of no return”: in case of need, hypothermia can be instituted rapidly, especially in low-weight babies.

When coupled to warm surgery, pulsatile perfusion generates a peripheral pulse detectable with an oximeter. As expected, during pulsatile warm perfusion, pulse oximetry values were equivalent to values measured with a co-oximeter. Several commercially available monitors display not only saturation but also a numerical value known as the perfusion index, a relative assessment of the pulse strength. This index is influenced mainly by the amount of blood at the monitoring site and not by the level of oxygenation and there is some evidence that it accurately reflects peripheral perfusion (99, 100). We routinely monitor peripheral saturation and perfusion index, peripheral saturation and perfusion index being likely to reflect saturation and perfusion quality of end organs. Furthermore, we use peripheral saturation to adjust FIO_2_ at the lowest level needed to reach a saturation of 98%, avoiding the potential deleterious effect of hyperoxia. In cyanotic patients, it was suggested that controlled reoxygenation decreased myocardial damage, oxidative stress, and cerebral and hepatic injury when compared to hyperoxic bypass (101). During aortic arch surgery, bilateral ear lobe sensor should display identical saturation and pulsatile index. Ear-lobe probe measures oximetry of the distal part of the external carotid artery and, in young patients, is likely to be a strong predictor of brain blood saturation. This simple tool could be a valuable alternative to monitoring of regional cerebral oxygenation via near-infrared spectroscopy. Studies comparing the two techniques are underway.

Beside the medical advantages, warm perfusion and intermittent warm blood microplegia are also cost-effective. The microplegia circuit is simple and therefore inexpensive and the arresting agent costs but a few euros. Furthermore, the negligible hydric balance of microplegia is a positive factor for blood conservation and contributes to reduce blood transfusion. For all these reasons, warm surgery was implemented by European teams in many humanitarian surgery missions. During missions, warm surgery is the perfect answer to the need for inexpensive and simple procedure, short time on bypass, and short intensive care length of stay, allowing more cases to be performed.

The main objection raised by opponents to warm surgery is the absence of safety margin in case of incident, requiring circulatory arrest lasting more than a few minutes. This is true, and if a surgeon experienced such incident during cold perfusion, he would probably better not implement warm surgery. However, after decades of application and tens of thousands of cases performed in numerous centers, we can confirm that the accidental risk is not a valuable reason to be afraid of warm surgery.

It is always challenging to choose a way opposite to the “gold standard” and the implementation of warm pediatric cardiac surgery was initially done against the opinion of the medical community. Nowadays, the results of pediatric cardiac surgery are so good that the number of patients needed to demonstrate the benefit of warm perfusion on mortality makes such a single center study utopian. Furthermore, cardiopulmonary bypass is only one element in the management of a patient. Cardiac surgery is a teamwork, the results of which depend on everyone involved in diagnosis, surgery, and intensive care including all the doctors, technician, and nurses from the medical staff. However, when a technique is simple, reproducible, safe, efficient, and cost-effective, and when no one changed his mind after choosing to implement it, it suggests that the approach makes sense. We can hardly imagine that full flow warm oxygenated blood brain perfusion could be worse than circulatory arrest or deep hypothermic regional cerebral perfusion. It is difficult to conceive that the good results observed in short-term outcomes on myocardial and brain functions could worsen over time, and we do hope that longitudinal studies will, in a next future, demonstrate the quality of long-term results of warm surgery.

## Author Contributions

The author confirms being the sole contributor of this work and approved it for publication.

## Conflict of Interest Statement

The author declares that the research was conducted in the absence of any commercial or financial relationships that could be construed as a potential conflict of interest.
